# Special Issue: “Genetics of Neuropsychiatric Disorders”

**DOI:** 10.3390/genes17080902

**Published:** 2026-07-30

**Authors:** Melloni N. Cook, Kristin M. Hamre

**Affiliations:** 1 Department of Psychology, The University of Memphis, Memphis, TN 38152, USA; 2 Department of Anatomy and Neurobiology, University of Tennessee Health Science Center, Memphis, TN 38163, USA

This Special Issue of *Genes*, entitled “Genetics of Neuropsychiatric Disorders,” collects articles advancing our understanding of these disorders and their genetic bases. Neuropsychiatric disorders are pervasive and can be debilitating; furthermore, individuals with these disorders often experience delayed diagnoses or present with comorbid disorders. Treatment options remain limited, are often associated with undesirable side effects, and have significant nonresponse rates. As many of these disorders involve a genetic component, identifying and characterizing related influences may help in discovering biomarkers for early/better detection, characterizing causal variants, and pinpointing novel pathways as targets for the development of therapeutics. This Special Issue includes studies on infantile seizures, sleep and mood disorders, stress-related disorders, schizophrenia, attention deficit/hyperactivity disorder, autism spectrum disorder and fetal alcohol spectrum disorders, highlighting the use of both human and animal models to identify the genes and pathways involved.

Neuropsychiatric disorders encompass a wide array of conditions, with some thought to have neurodevelopmental origins. Nervous system development is a protracted process that begins early in the first trimester of gestation and continues through adolescence and into early adulthood. The manifestation of neuropsychiatric disorders can occur at any time throughout this timeframe. As many as 15% of children are diagnosed with a neurodevelopmental disorder [1], with the evidence supporting a link between neurodevelopmental and adult psychiatric disorders [2]. The articles in this Special Issue analyze the genes and genetic pathways that underlie the development of disorders within this time span.

Jang and colleagues [3] used whole-exome sequencing to identify a mutation in a patient with infantile epileptic spasms, a type of epilepsy that typically first occurs within the first year of life and is characterized by intense muscle contractions. The mutation was shown to be present in the calponin homology domain 2 of the *SPTBN1* gene, which encodes the cytoskeletal protein BetaII Spectrin. Mutations in *SPTBN1* have been associated with other types of epilepsy as well as other types of NDs. These associations were cataloged following an extensive literature review to provide a more complete picture of the role of *SPTBN1* in neurodevelopment. More importantly, improving our understanding of the genetic basis of infantile epileptic spasms will support the development of precision medicine options [4].

Adolescence is a period when both sleep problems and mood disorders become apparent. Goodell and Ingram’s study of university students [5] explored the relationship between SNPs in the circadian rhythm gene, *Per3*, and a range of phenotypes—sleep patterns, anxiety, depression, internalizing disorders, and seasonal affective disorders—as measured via self-reporting questionnaires. Specific SNPs were associated with specific phenotypes, some of which were sex-specific. Interestingly, greater associations were found in the analysis of multi-SNP haplotypes, emphasizing the importance of evaluations conducted across haplotypes. Although the mechanisms are not known, numerous polymorphisms in genes that belong to the Clock family of genes have been associated with psychiatric disorders [6].

Stress-related disorder (SRD) is a classification that encompasses a host of disorders such as adjustment disorders and post-traumatic stress disorder. The onset of these disorders is typically thought to require both exposure to a stressor and a genetic predisposition. While the genetic underpinnings in adults have been the focus of numerous studies, less emphasis has been placed on childhood SRDs and their underlying genetic factors. Raffagnato and colleagues [7] used literature searches and data collation to identify the pathways most often associated with childhood SRDs, identifying critical pathways that conferred enhanced likelihood of developing an SRD after stress exposure as well as pathways that provided resilience to stress exposure. Given their relationship to adult psychopathology, it is vital to improve our understanding of childhood stress-related disorders [8].

A more targeted approach was used by Kowalczyk et al. [9], who examined polymorphisms in the heat shock protein 90 alpha, encoded by *HASP90AA1*, in Polish individuals (both male and female) that were diagnosed with schizophrenia compared to controls. None of the SNPs demonstrated an association with schizophrenia either in the population as a whole or when compared within sexes. However, specific SNPs did show an association with the emotional distress dimension score, which evaluates negative affect in an individual and is often related to treatment outcome.

Animal models can provide insights into potential therapeutic agents and the gene pathways they work within. Choline may have therapeutic potential for the treatment of fetal alcohol exposure; supplementation has been shown to reduce ethanol-induced cell death in embryos from a subset of the BXD RI strains [10]. Given the early period (E 9.5) evaluated and the aforementioned strain differences, it is most likely that maternal choline metabolic genes are related to choline’s ability to reduce cell death; therefore, the role of genetics in choline metabolism was examined. Using bioinformatics tools, Chowdhury et al. [11] developed a list of 22 choline metabolic genes for consideration. After reviewing haplotype analysis; examining liver protein expression and correlations between liver protein expression and cell death; and reviewing the literature and human GWAS SNPs, the list was pared down to two potential candidate genes related to choline metabolism: choline/ethanolamine phosphotransferase 1 (*cept1*) and choline transporter gene solute carrier family 44 member 1 (*slc44a1*). These were the only genes demonstrating a relationship between protein expression in the liver and cell death in the forebrain and brainstem. Further, SNPs associated with these genes were associated with developmental disorders (*cept1*) and alcohol use disorders (*slc44a1*). Importantly, genetic differences in the maternal metabolism of choline likely affect the efficacy of supplementation.

Bajpai et al. [12], also using the BXD RI strains, examined prepulse inhibition, a sensory motor gating trait related to schizophrenia. Mice from 59 BXD RI strains were screened for acoustic startle and prepulse inhibition. Using genetic mapping, a significant male-specific quantitative trait locus (QTL) was identified on chromosome 19 for % prepulse inhibition at the 85dB level (%PPI 85dB). ATPase family, AAA domain-containing 1 (*Atad1*) was identified as a strong candidate gene related to this trait. Bioinformatic tools allowed the authors to identify genes correlated with *Atad1*, many of which were related to neurotransmission. Further analyses suggest that the protein ATAD1 likely works through its direct partners, GRIA2 and ASNA1. Human PheWAS data revealed associations between *Atad1* and psychiatric traits, while human RNA-seq data showed differences in *Atad1* expression between controls and patients with schizophrenia. Collectively, these data provide strong support for the role of *Atad1* in schizophrenia-related traits.

Over the past 20 or so years, animal models have been at the forefront of advances in our knowledge of attention deficit hyperactivity disorder (ADHD) and autism spectrum disorders (ASD) [13]. Brewer et al. [14] examined inattention in two different rat models of attention deficit hyperactivity disorder (ADHD). Event-related potentials (ERPs), which measure automatic attention, were evaluated in latrophilin-3 (*Lpn3*) knockout (KO) rats and spontaneously hypertensive rats (SHR). The *Lphn3* KO rats did not show abnormal attention; however, the SHR rats displayed reduced P1 and P1-N1 events, indicative of deficits in automatic attention. The differences in ERPs in the *Lphn3* KO and SHR models are likely due to differences in the underlying brain mechanisms in the two models. As inattention has been understudied, this study highlights the potential usefulness of ERPs in examining ADHD-related traits.

Pal et al. [15] examined conditioned taste aversion in an animal model of autism spectrum disorder (ASD). Many individuals with an ASD are thought to be “picky” eaters and/or have a limited diet; however, it is unclear whether this “pickiness” is due to avoiding novel foods or taste aversion. When given during pregnancy, valproic acid (VPA) can increase the risk of ASD. Rats given VPA showed the expected open-field and elevated-plus-maze behaviors but did not display conditioned taste aversion. VPA rats were given saccharin solutions then administered varying doses of LiCl. Lithium failed to induce the expected conditioned taste aversion in VPA rats; however, feeding-, stress-, and anxiety-related genes were found to be differentially regulated in their brains. The results suggest that although the VPA rats did not display conditioned taste aversion, they likely developed impaired associative learning.

The work presented in this Special Issue has identified an *SPTBN1* mutation associated with infantile epileptic seizures, the pathways involved in childhood stress disorders, and the role of maternal choline metabolic genes in the efficacy of choline supplementation as a treatment for fetal alcohol exposure. Additionally, the roles of specific HSPA90AA1 SNPs in schizophrenia, the *Per3* clock gene in sleep-related disorders, *Atad1* as a strong potential candidate for schizophrenia-related traits, and feeding- and anxiety-related genes in a valproic acid rat model of autism spectrum disorders have all been examined. Lastly, inattention was evaluated as a potential biomarker of ADHD in two rat models. The work presented in this Special Issue shows that genetic factors can influence a range of neuropsychiatric disorders across the lifespan and that even the genetics of the maternal uterine environment can exert effects on the development of some disorders. Because of conceptual, technical, and bioinformatic advances, we are better positioned to understand these complex disorders involving many genes. If we are able to better understand the contributions of genes and the interactions between them, this information can eventually be applied to the development of better diagnoses and treatments [16].
